# The Effect of Stocking Density and Carbon Sources on the Oxidative Status, and Nonspecific Immunity of *Nile tilapia* (*Oreochromis niloticus*) Reared under Biofloc Conditions

**DOI:** 10.3390/ani11010184

**Published:** 2021-01-14

**Authors:** Ramy M. Shourbela, Shymaa A. Khatab, Mohamed M. Hassan, Hien Van Doan, Mahmoud A. O. Dawood

**Affiliations:** 1 Department of Animal Husbandry and Animal Wealth Development, Faculty of Veterinary Medicine, Alexandria University, Edfina 22758, Egypt; ramy_aqua@yahoo.com; 2 Genetics and Genetic Engineering, Department of Animal Husbandry and Animal Wealth Development, Faculty of Veterinary Medicine, Alexandria University, Edfina 22758, Egypt; shymaa.khattab@alexu.edu.eg; 3 Department of Biology, College of Science, Taif University, P.O. Box 11099, Taif 21944, Saudi Arabia; m.khyate@tu.edu.sa; 4 Department of Genetics, Faculty of Agriculture, Menoufia University, Sheben El-Kom 51132, Egypt; 5 Department of Animal and Aquatic Sciences, Faculty of Agriculture, Chiang Mai University, Chiang Mai 50200, Thailand; 6 Innoviative Agriculture Research Center, Faculty of Agriculture, Chiang Mai University, Chiang Mai 50200, Thailand; 7 Department of Animal Production, Faculty of Agriculture, Kafrelsheikh University, Kafrelsheikh 33516, Egypt; 8 Center for Applied Research on the Environment and Sustainability, The American University in Cairo, New Cairo 11835, Egypt

**Keywords:** biofloc, carbon source, stress markers, *Nile tilapia*, stocking density, gene expression

## Abstract

**Simple Summary:**

The present study investigated the effect of stocking density and dietary carbon sources on the water quality, oxidative status and immune-related of Nile tilapia (*Oreochromis niloticus*) reared under biofloc conditions (BFT). Eight groups were established at two levels of stocking densities (140 fish per m^3^: low stocking density, LSD) and (280 fish per m^3^: high stocking density, HSD) (5.15 ± 1.12 g) and kept in eight biofloc units containing water without carbon sources (control groups) or with glycerol, molasses, or starch. Overall, this study has reported that immune response gene expression is better in LSD than HSD and improved by carbon addition. More specifically, based on the overall performances of tilapia reared under LSD or HSD, using molasses is recommended as a carbon source to promote the performances and health status of Nile tilapia cultured in a biofloc system.

**Abstract:**

The present study investigated the effect of stocking density and dietary carbon sources on the water quality, oxidative status, and immune-related genes of Nile tilapia (*Oreochromis niloticus*) reared under biofloc conditions (BFT). Eight groups were established at two levels of stocking densities (140 fish per m^3^: low stocking density, LSD) and (280 fish per m^3^: high stocking density, HSD) (5.15 ± 1.12 g) and kept in eight biofloc units containing water without carbon sources (control groups) or with glycerol, molasses, or starch. Red blood cells count, hemoglobin, and hematocrit values were reduced in fish stocked in control groups at LSD and HSD than biofloc groups. Control fish groups reared at both LSD and HSD have the highest significant (*p* < 0.05) white blood cells number than other fish groups. Meanwhile, fish groups that received glycerol, molasses, and starch maintained in both LSD and HSD presented a higher significant (*p* < 0.05) monocyte % than in the control group reared at both LSD and HSD. The fish group reared in biofloc conditions (BFT) using starch carbon source and reared at the HSD presented a significantly higher (*p* < 0.05) increase in total serum protein and albumin levels as well as globulin value than the control fish group reared at both LSD and HSD. The highest glucose and cortisol levels were showed in the control fish group reared at both LSD and HSD. Fish maintained in glycerol-based biofloc at LSD attained the highest (*p* < 0.05) serum superoxide dismutase (SOD), glutathione reductase (GR), and catalase than other experimental groups. Regarding the nonspecific immune status, significantly increased expression of CC-chemokines, CXC-chemokines, TLR7 and IL-8 genes was found in molasses based biofloc groups. The data of the present study revealed that using molasses promotes health status of Nile tilapia cultured in a biofloc system.

## 1. Introduction

In recent decades, recirculating systems have been developed in the course of sustainable aquaculture, involving an appropriate approach to control aquaculture wastewater [1]. In this system, 10% of the total volume of water is replaced daily, but due to the high operating and maintenance costs, adoption of the recirculating system among farmers, especially in developing countries, is low [2]. Therefore, the need for a low-cost, sustainable, and environmentally friendly technology that is acceptable to farmers and can be used on a large scale is evident and noteworthy.

The biofloc system, also called biofloc technology (BFT), has recently attracted great attention as a cost-effective, sustainable, and environmentally friendly (as the water exchange rate and artificial feeding ratio are reduced) way that improves water quality as well as produces microbial protein for aquatic species [3,4]. BFT is a technique of enhancing water quality by adding extra external carbon sources in accordance with a high level of aeration to produce high levels of microbial bacterial floc in the aquaculture system [5]. Bioflocs are rich sources of nutrients, and their level of intake depends on fish’s feeding habits and their behavior in utilizing the flakes. In this regard, Nile tilapia (*Oreochromis niloticus*) is known for the omnivorous feeding habit, which allows them to utilize BFT efficiently as a source of protein and carbohydrates [6]. BFT contains about 30% crude protein, covering up to 50% of tilapia’s protein needs and protein sparing of aquafeed [7,8]. Furthermore, BFT offers a natural probiotic effect through the presence of beneficial microorganisms and their cell components, which act as growth-promoting and immunostimulant factors. Accordingly, BFT results in enhanced growth performance, immune response, antioxidative status, and resistance to aquaculture stressors [9].

Maintaining a carbon-to-nitrogen ratio is essential in the BFT system by adding carbon-containing organic materials such as molasses, wheat flour, or starch, or reducing the protein level of the feed to increase the activity of heterotrophic bacteria [10]. Different types of organic carbon sources (e.g., glucose, acetate, starch, wheat, glycerol, molasses etc.) determine to a large extent the degree of the composition of flocs produced, mainly regarding the type and amount of storage polymers [11]. The implementation of BFT in tilapia has been successfully applied using different carbon sources. The overall results illustrated that applying BFT resulted in improving the water quality and the growth performances of Nile tilapia without compromising the health condition [12,13]. However, little effort was made to study the effect of BFT under different stocking densities on physiological health and non-specific immunity of Nile tilapia [14]. Additionally, very few studies investigated the immunological potential of the biofloc technology.

Therefore, this study aimed to investigate the application of BFT using different carbon sources for Nile tilapia maintained under different stocking densities on some hematological and biochemical indices, antioxidant status as well as the nonspecific immune response and immune-related genes of Nile tilapia.

## 2. Materials and Methods

### 2.1. Experimental Design

*Nile tilapia* were obtained from a private farm at Kafr Elsheikh governorate, Egypt. The fishes were acclimatized in plastic tanks (500 L × 3) for 14 days and were fed with a commercially produced floating pelleted feed (Hendrix Feed Co., Belbeis, Egypt) having a protein content of 30% approximately. Also, during the acclimatization period, any fish showing apparent signs of disease or malnutrition were separated out. Fish of mean weight of 5.15 ± 1.12 g were randomly distributed into 24 tanks to form eight experimental groups in triplicate following a completely randomized design. All the experimental fishes were fed twice a day between 08:00 and 09:00, and 18:00 and 19:00.

The experiment was conducted for 98 days in 24 plastic circular tanks (500 L) with two different densities (low stocking density “LSD, 140 (5.15 ± 1.12 g) fish per m^3^” and high stocking density “HSD, 280 fish per m^3^”) and four different carbon sources as: control (no extra organic carbon addition), glycerol, molasses, and starch. The stocking densities of tilapia were proposed by following Dawood et al. [15] and Ran et al. [16], where each m^3^ was stocked with 1442 g and 2884 g, respectively. In BFT treatments, the water was maintained at the optimum level by including freshwater daily at about 10% for the water shortage compensation due to evaporation [2]. Further, the water of the control groups was exchanged daily with dechlorinated water. The water quality indices in all tanks were measured and the values were 7.2 ± 0.3, 0.12 ± 0.1 mg/L, 0.12 ± 0.1 mg/L, 0.5 ± 0.1 mg/L, and 1.2 ± 0.5 mg/L for pH, ammonia nitrogen (NH_4_-N), nitrite nitrogen (NO_2_-N), and nitrate nitrogen (NO_3_-N), accordingly using Jenway spectrophotometer 3010 multi-parameter analyzers using environmental test kits: JENW 025 303 for un-ionized ammonia, JENW025326 for nitrite, JENW 025 325 for nitrate.

### 2.2. Biofloc Initiation

The formation of inoculums flocs was done in plastic containers one week before the initiation of the trial by adding 20 g of pond bottom soil in well-aerated water (1 L) with 10 mg/L urea and 400 mg/L of different carbon sources (glycerol, molasses, and starch) and 20 g yeast. The tanks were prepared to promote biofloc production by filling the tanks to 30% capacity and the prepared inoculums were added to their respective experimental groups. When the tanks contained 0.2 mL/L concentration of biofloc bacteria, juvenile tilapia were placed in the tanks, and the tanks were filled with water.

A 1.5 kW side channel blower was used and ran continuously throughout the duration of the experiment. It fed into a 40 mm grey colored irrigation piping, which ensured that each individual tank received reasonable aeration and mixing. To ensure proper agitation and mixing of the floc as well as to meet the oxygen demand, from the 40 mm ring, porous rubber hoses and four air stones connected to the main aeration pipe by 15 mm flexible pipes were supplied to each tank. The C:N ratio was maintained at 15:1 for the development of biofloc. The daily quantity of carbon added was calculated according to Avnimelech [17] and Avnimelech and Kochba [18]. Carbon sources were added twice a day between 08:00 and 09:00, and 18:00 and 19:00.

### 2.3. Hematology and Non-Specific Immune Responses

Blood samples of five fish per tank were collected randomly from the caudal vein at the end of the trial, 24 h after the last feeding. For blood sampling, fish were anaesthetized with buffered MS-222 (Sigma Aldrich, St Louis, MA, USA). They were wiped and cleaned to avoid mucus mixing into the blood. Blood was collected by a 3 mL plastic syringe, irrigated with sodium citrate, without harming fish according to Val, et al. [19]. Then, 1.5 mL blood was transferred to ethylene diamine tetra acetic acid (EDTA) tubes for hematological analysis. The other 1.5 mL of blood was transferred to plastic biochemistry test tubes. After blood was coagulated, tubes were centrifuged at 3500 rpm for 15 min for serum separation, which was stored below −20 °C according to Blaxhall and Daisley [20].

#### 2.3.1. Hematological Analysis of Whole Blood

Total red blood corpuscles (RBCS) and total leukocytic counts (WBCS) were determined by hemocytometer according to Maxine and Benjamine [21]. Hemoglobin content (Hb) was determined by Jenway spectrophotometer multi-parameter analyzers using Drabkin’s reagent according to van Kampen and Zijlstra [22]. The leukocytic count was determined through an indirect method using blood smear stained with May–Grunwald–Giemsa, according to the method described by Lucky [23] for differential leukocytic count, and absolute values for each type of cells were calculated according to Schalm [24].

#### 2.3.2. Immunological and Biochemical Analysis of Serum

Biochemical indices in serum were measured using Jenway spectrophotometer multi-parameter analyzers using Bio-analytic test kits (Biodiagnostic Industry, Co. Giza, Egypt). Total protein and albumin were respectively determined by the biuret and the bromocresol green (BCG) reactions as described elsewhere [25,26]. Globulin levels were calculated after subtracting albumin from total protein content, and the albumin to globulin (A:G) ratio was calculated. The enzymatic colorimetric method, glucose oxidase-phenol amino phenazone (GODPAP), was used to assay the profile of serum glucose [27]. Serum cortisol levels were determined using electro chemiluminometric assay by Elecsys and Cobas e 411 Immunoassay Analyzer (Roche Diagnostics, Indianapolis, IN, USA). The test kit was prepared in accordance with the method described by Chiu et al. [28].

##### Assay of Serum Superoxide Dismutase (SOD) Activity, Glutathione Reductase (GR), Catalase (CAT) Activity and Nitric Oxide (NO) Activity

The serum antioxidants were measured using Jenway spectrophotometer multi-parameter analyzers. Superoxide dismutase (SOD) and nitric oxide (NO) activity was evaluated using commercial kits (Spinreact kits, Sigma Industry, Co. Girona, Spain) based on the methods described in the instructions [29,30], while glutathione reductase (GR) was measured according to the method described by Goldberg and Spooner [31]. Catalase activity in blood serum was measured according to Aebi [32].

### 2.4. Gene Expression Analysis

#### 2.4.1. Isolation of RNA and cDNA Synthesis

Five fish from each group were used to collect spleen tissue and immediately the tissue was placed in 2 mL Eppendorf and frozen in liquid nitrogen then kept at −80 °C for RNA isolation. Total RNA was extracted by using (Trisure reagent)^®^ according to the manufacturer’s instructions (iNtRON Biotechnology, Inc., Seongnam, Gyeonggi, Korea). Extracted RNA was stored at −80 °C. Total RNA was reverse transcribed into cDNA by using a power first strand cDNA synthesis kit (Intron, Korea) according to the manufacturer protocol. The obtained cDNA was stored at −20 °C until further use. The cDNA was checked by housekeeping gene (β-actin), then the product was checked on 2% agarose gel electrophoresis.

#### 2.4.2. Quantitative Real Time Polymerase Chain Reaction (qRT-PCR)

Quantitative real time PCR for the pro inflammatory-related genes as interleukin 8 (IL-8), CC-chemokine, and C-X-C chemokine and immune-related genes as Toll-like receptor7(TLR7) were done in qPCR machine (3000× Stratagene, Bioline/Meridian Bioscience, London, UK). qRT-PCR reaction was performed by using SensiFastTM SYPER Low-Rox kit (Enzynomic, USA). Primers annealing temperature and PCR products are listed in Table 1. In 0.2 mL PCR tubes, the following ingredients were included, 0.8 µL of each forward and reverse primer, 2 µL of cDNA, 6.4 µL RNAase free water and 10 µL SYPER green. The thermal cycler condition was one cycle of denaturation at 95 °C for 10 min followed by 40 cycles of 95 °C for 15 s and annealing temperature 60 °C for one min. The dissociation curve was generated at the end of the last cycle by collecting the fluorescence data at 60 °C and taking measurements every 7 s until the temperature reached 95 °C to validate the specificity of the PCR amplicons. To ensure that a signal product was amplified, melting curve analysis was performed on the PCR products at the end of each PCR run. The β-actin was used as a housekeeping gene. The analysis of gene expression was performed by comparative threshold cycle method 2^−ΔΔct^ [33].

### 2.5. Statistical Analysis

Normality and homoscedasticity analyses were adopted before applying a one-way ANOVA method, and then Duncan’s multiple-range test was used to determine differences among treatments. Differences in the hemato-biochemical indices due to stocking density and BFT system (using different carbon sources) were determined. The data were analyzed using two-way analysis of variance by applying SAS (2004) software. Significance level was established at *p* < 0.05. Data analysis of gene expression was done by one-way ANOVA. Statistical analysis was done using GraphPad Prism software version 6 (Graph prism Software, La Jolla, CA, USA). The results were obtained as mean ± standard error (SE).

## 3. Results

### 3.1. Hematology and Immune Response of Fish

#### 3.1.1. Fish Hematological Parameters

In this study, the addition of different carbon sources, rearing densities, and their interaction significantly affected red blood cell count (RBCs), hemoglobin (Hb), packed blood volume (PCV), white blood cell count (WBCs), monocyte, lymphocyte, and eosinophil of Nile tilapia (Table 2). RBCs, Hb, and PCV were positively affected by the addition of different carbon sources in the BFT system. Fish group received glycerol, molasses and starch as a carbon source and maintained in both LSD and HSD presented significantly higher (*p* < 0.05) RBCs, Hb, and PCV values than in control group maintained in both LSD and HSD (Table 2). The control group reared at both LSD and HSD had significantly lower (*p* < 0.05) RBCs (2.74 ± 0.38 and 2.50 ± 0.32 × 10^6^ μ/L) than all groups. The lowest Hb and PCV values were found in fish reared at both LSD and HSD of the control groups. Glycerol-based BFT and maintained in LSD had the highest significant (*p* < 0.05) PCV value (46.4 ± 6.80 %) than control and other BFT treatment.

Control fish groups reared at both LSD and HSD have the highest significantly (*p* < 0.05) WBCs number than other fish groups (126,520 and 107,200 μ/L). Fish of the control groups and reared in HSD showed a significantly higher lymphocyte (9.4 ± 4.39%) compared to other treatments. Meanwhile, the fish group that received glycerol, molasses, and starch and was maintained in both LSD and HSD presented a higher significant (*p* < 0.05) monocyte % than in the control group reared at both LSD and HSD. Eosinophil % showed no significant difference among fish groups.

#### 3.1.2. Serum Biochemical Analyses

Serum biochemical parameters of Nile tilapia reared under different stocking densities using different carbon sources are presented in Table 3. Serum biochemical analyses were significantly affected by different carbon sources, rearing densities, and their interaction. The fish group reared on BFT using starch carbon source and reared at the HSD presented a significantly higher (*p* < 0.05) increase in total serum protein (5.73 ± 0.42 g/dL) and albumin (1.79 ± 0.28 g/dL) levels as well as globulin (3.94 ± 0.25 g/dL) values than the control fish group reared at both LSD and HSD. The control fish group reared at HSD had a lower total protein (3.82 ± 0.74 g/dL), albumin (1.19 ± 0.30 g/dL) and globulin (2.63 ± 0.78 g/dL) than other fish groups that received different carbon sources at both stocking densities (LSD and HSD), while A/G ratio showed no significant difference among fish groups.

Stress indicators like serum cortisol and serum glucose were lower in all BFT-based treatments than control treatments at both LSD and HSD. Concerning glucose level, there is an interaction between different carbon sources and rearing stocking density. The highest glucose levels were shown in control fish groups reared at both LSD and HSD (144.4 ± 19.68 and 139.8 ± 13.97 mg/dL, respectively), while BFT-based treatments at both densities showed a significantly lower glucose value than control groups, moreover the glycerol-based BFT group reared at HSD showed the lowest glucose level among all experimental groups.

Serum cortisol level varied significantly among the different treatments, where BFT-based treatments had lower cortisol levels than control groups. The lowest cortisol levels were presented in glycerol based BFT groups reared at both LSD and HSD (6 ± 0.85 and 8.36 ± 4.58 ng/dL, respectively), while the highest levels were shown in the control fish group and reared at both LSD and HSD (41 ± 5.57 and 37.6 ± 10.53 ng/dL).

#### 3.1.3. Antioxidant in Serum

Serum antioxidant levels were significantly affected by different carbon sources addition, rearing densities, and their interaction (Table 4). Fish maintained in glycerol based BFT at LSD attained the highest (*p* < 0.05) serum superoxide dismutase (SOD) (62.4 ± 9.79 U/L), glutathione reductase (GR) (43.4 ± 7.4 U/L) and catalase (44.6 ± 4.28 U/L) than other experimental groups. Meanwhile, control fish groups reared at HSD showed a significantly (*p* < 0.05) lower serum SOD (26 ± 3.39 U/L), GR (24.2 ± 6.61 U/L), and catalase (19.4 ± 5.22 U/L) than other groups.

On the other hand, serum nitric oxide displayed significantly (*p* < 0.05) elevated levels in the control fish group raised at LSD and HSD (23.52 ± 6.36 and 24.4 ± 7.84 U/L, separately) more than BFT-based groups, whereas the lowest significant (*p* < 0.05) nitric oxide level was presented in glycerol based-BFT and reared at LSD.

### 3.2. Gene Expression

#### 3.2.1. Low Stocking Density (140 Fish in m^−3^)

**Figure 1:**
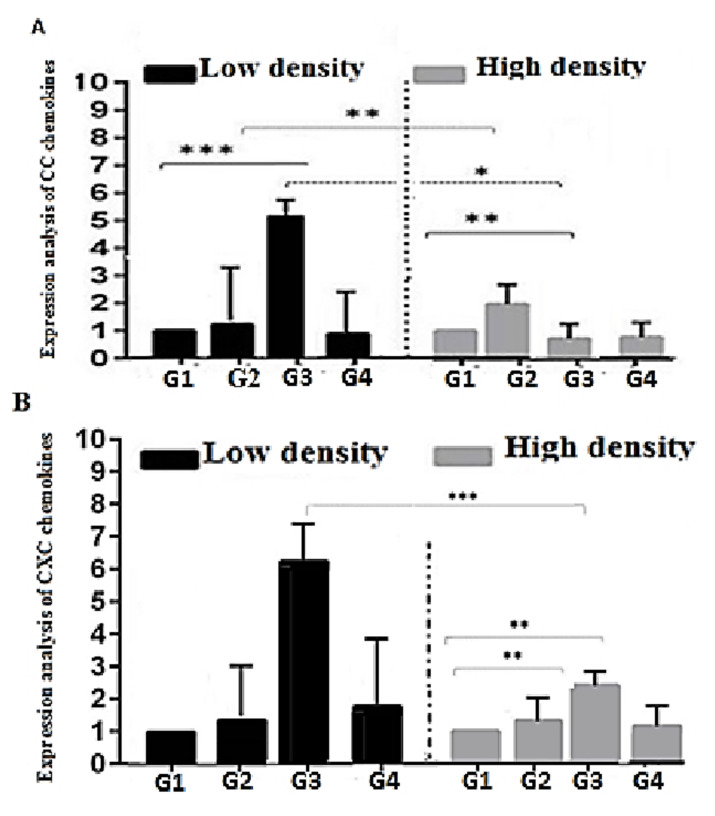
Relative expression of some inflammatory-related genes (CC-chemokines (**A**) and CXC-chemokines (**B**)) in Low and High stocking density of Nile tilapia (*Oreochromis niloticus*) with different treatment. All values are expressed as Mean ± SE. Asterisks on the data bars indicate when *p* < 0.05 (*), *p* < 0.005 (**), and *p* < 0.0005 (***). G1 (control without carbon source), G2 (treated with glycerol as a carbon source), G3 (treated with molasses as a carbon source), and G4 (treated with starch as a carbon source).

**Figure 2:**
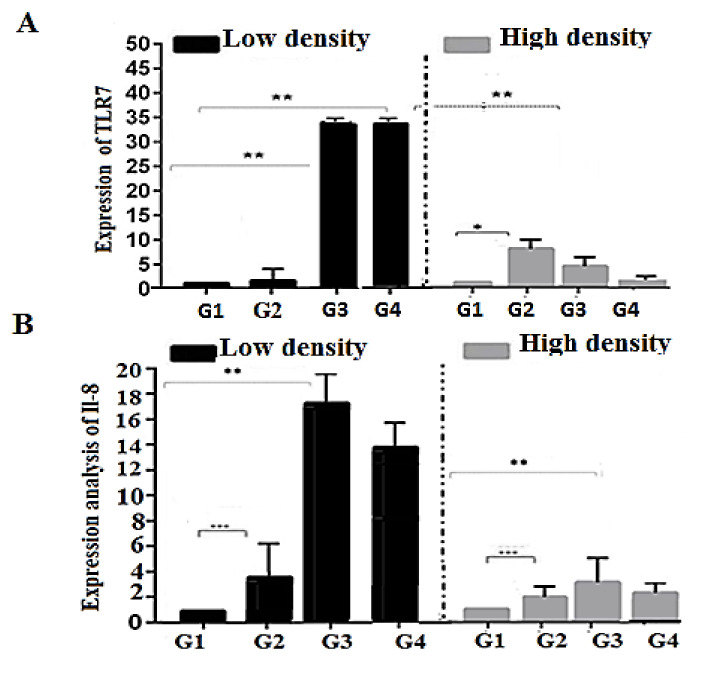
Relative expression of some immune response genes TLR-7 (**A**) and IL-8 (**B**) in Low and High stocking density of Nile tilapia (*Oreochromis niloticus*) with different treatment. All values are expressed as Mean ± SE. Asterisks on the data bars indicate when *p* < 0.05 (*), *p* < 0.005 (**), and *p* < 0.0005 (***). G1 (control without carbon source), G2 (treated with glycerol as a carbon source), G3 (treated with molasses as a carbon source), and G4 (treated with starch as a carbon source).

LSD in the group treated with glycerol had no effect on the CC-chemokines and CXC-chemokines expression, as they were maintained within the control level. Mild up-regulation of TLR7 gene to two folds was recorded, and was also associated with a significant up-regulation of inflammatory related genes IL-8 to 4 folds. Regarding the effect of molasses, the results showed a significant up-regulation of both CC-chemokines, CXC-chemokines, TLR7, and IL-8 to 5, 6, 34, and 18 folds, respectively. The addition of starch induced up-regulation of CXC-chemokines, TLR7, and IL-8 to 2.33 and 14 folds, respectively. Meanwhile, it is associated with a significant decrease of CC-chemokines to 0.8 folds (Figure 1 and Figure 2).

In comparison of different treatments in LSD, molasses was associated with the highest up-regulation of all immune response genes followed by starch, while glycerol showed the lowest expression.

#### 3.2.2. High Stocking Density (280 Fish in m^−3^)

The BFT based group treated with glycerol and stocked with HSD showed a significant up-regulation of CC-chemokines and TLR-7 gene to 2 and 7 folds, and mild significant up-regulation of CXC-chemokines and IL-8 gene to 1.2 and 2.3 folds (Figure 1 and Figure 2). Regarding the effect of molasses, the results showed a significant up-regulation of all genes: CXC-chemokines, TLR7, and IL-8 to 2.4, 5.5, and 3.5 folds, respectively, while it caused down-regulation of CC-chemokines to 0.68. BFT based on the starch group showed down-regulation of CC-chemokines and TLR7 to 0.73 and 0.58 folds (Figure 1 and Figure 2). Meanwhile it is associated with an increase of IL-8 to 2.5 folds, without any effect on CXC-chemokines gene expression.

In comparison of different treatments in HSD, the effect of glycerol was associated with the up-regulation of all immune response genes (CC-chemokines, CXC-chemokines, TLR7, and IL-8), while molasses showed an increased expression of genes (CXC-chemokines, TLR7 and IL-8) and down regulation of CC-chemokines gene and the starch showed the lowest expression.

## 4. Discussion

In intensive aquaculture, where high stocking densities and little water exchange is applied, ammonia build-up from feed metabolism is generally the limiting factor on fish growth and wellbeing [39]. Bacteria, bacterial products, carbohydrates, and nutritional factors play a key role in immune-stimulatory action in normal cases [40]; the presence of the same in BFT may have contributed to enhanced immunity of the culture organisms [41]. The juveniles of Nile tilapia reared on BFT using glycerol, molasses, and starch as carbon sources at both LSD and HSD presented a significant increase in red blood cell count (RBCs), hemoglobin (Hb), and packed blood volume (PCV) values. Similarly, an enhanced effect of BFT on RBCs and Hb was recorded in common carp fingerlings [42], *Labeo rohita* [43], and shrimp [44]. The immune system activation is carried out by increasing involved molecules in their routes in which there are a lot of cell types (e.g., monocytes and phagocytes) and molecules involved in very different immune pathways that could be related to oxidative stress and inflammation [45,46]. In contrast to our results, no significant difference was observed between the control and BFT system in RBCs and hematocrit in tilapia [47], *Farfantepenaeus brasiliensis* [48], and *Litopenaeus vannamei* [49]. Additionally, the application of a BFT system enhanced the biochemical parameters (total serum protein, albumin, and globulin). The possible stressful conditions to the fishes in BFT system can be ruled out by the serum cortisol and glucose level. Increased levels of serum cortisol and serum glucose are reported in Nile tilapia when exposed to different kinds of stress [50,51]. In the present study, cortisol, and serum glucose in all biofloc treatments were lower than in control groups at both rearing densities. This indicates that the BFT unit does not cause any stress-related problems to the fish even at stocking densities up to (280 fish/m^3^). Also, the reduced levels of cortisol are probably attributed to the high content of BFT of nutrients, vitamins, and beneficial substances that fulfilled the nutritional requirements of tilapia reared under the LSD and HSD conditions. The results are comparable with earlier findings in Nile tilapia [47] and in *Labeo rohita* [43]. Also, Haridas et al. [52] reported significantly lower levels of serum cortisol and glucose for tilapia maintained in BFT units (SD1 and SD2) compared to the control.

The nitric oxide (NO) activity has been used as a nonspecific immune response in several stressed fish species e.g., common carp [53] and rainbow trout [54]. Several potent oxidizing reactive nitrogen intermediates (such as peroxinitrite) that are produced from the reaction between NO and reactive oxygen intermediates (such as superoxide) can efficiently kill pathogens [55]. In the present investigation, the BFT system substantially decreased the activity of NO as the rearing density increased. This result plus the recorded reduced glucose and cortisol levels gives further support to the effect of BFT as a stress relieving effect in intensively cultured fish. On the other hand, the reduced NO activity detected in the BFT could be due to the generous source of many bioactive compounds (e.g., chlorophylls, carotenoids, phytosterols, bromophenols, and amino sugars) [56] and antibacterial compounds [41] of the biofloc which helps to reduce the density related stress and positively influences the robustness of the fish immune system. Additionally, biofloc is known for its beneficial probiotic effect, which could act internally and/or externally against pathogenic *Vibrio* sp. and ecto-parasites in BFT, respectively [57]. Similar results were conveyed by El-Hawarry, et al. [58] where they recorded increased NO activity in juvenile tilapia maintained in HSD.

Antioxidant enzymes are frequently used as an indicator to oxidative stress resulting from pathogen pressure and environmental perturbations, and therefore can reflect the animal health status [59,60]. Three classes of antioxidative enzymes (superoxide dismutase, catalase, and glutathione reductase) are associated with the prevention of lipid peroxidation. SOD catalyzes superoxide anion to produce hydrogen peroxide [61] which, in turn, is decomposed by CAT to water and oxygen, preventing the beginning of lipid peroxidation. Referring to this study the LSD glycerol based BFT group showed a marked increase in the levels of SOD, GR, and CAT as compared to the HSD non-BFT based fish group. Several studies showed that the application of BFT system also improved antioxidant activity of the cultured animals [60]. Luo et al. [62] demonstrated that the serum total SOD activity in BFT grown tilapia was significantly higher than in the fish cultured in the recirculating aquaculture systems (RAS). Likewise, Xu and Pan [60] reported that the total antioxidant activities of shrimp in BFT groups were more than two times higher than that of the control (clear water). Xu and Pan [60] suggested that this antioxidant activity enhancement effect of BFT might relate to the contribution of some bioactive compounds known to have antioxidant effects such as carotenoids, vitamin C, and essential fatty acids. In addition, it should be noted that stocking density plays a crucial role in this prospect. It is obvious that the HSD glycerol based BFT group showed an impressively lower serum SOD and CAT than the LSD one. This result is certainly matched with the result conveyed by El-Hawarry et al. [58] where they recorded a reduced oxidative stress as long as the tilapia density increased from 100 fish m^−3^ to 400 fish m^−3^ when fed a diet supplemented with essential oil that is known with its antioxidant bioactive compounds.

Raising fish under HSD is among the main stressors that led to the deterioration of fish performances because of impaired immunity [15]. Concurrently, the detection of the immune and pro-inflammatory related genes is a vital tool to express fish’s status under high stocking density. The expression of the pro-inflammatory and immune related genes (IL-8, CC-chemokine, and C-X-C chemokine) is associated with the fish’s response to cope with the stress features. The response includes several functions: promoting adherence of neutrophils to endothelial cells, neutrophil chemo-attractant ability, histamine release, and subsequent migration toward inflammatory sites [63]. Likewise, the expression of TLR7 is related to the antistress response of fish to relieve inflammation impacts [64]. The LSD groups with different carbon sources affected the immune response genes, where TLR-7 and IL-8 were highly upregulated to 34- and 18-fold increase of molasses. Additionally, to 33- and 14-fold on starch treatment, but the increase of glycerol expression of TLR7 was around control and IL-8 slightly increased up to 4-fold. We suggested that increased expression of immune response genes (TLR7 and IL-8) occurs simultaneously in normal health condition related to the spleen as an immune response organ, this is in agreement with Qian, et al. [65] who approved that the TLR7 was constitutively expressed in immune-related tissues especially in spleen, gills, and head kidney. Similarly, IL-8 is expressed in many tissues in fish under normal conditions [66]. Yu, et al. [67] reported that decreased expression of IL-1β and TNF-α indicated that BFT could enhance immunity of golden crucian carp. Additionally, the results of immune-related genes agree with Menaga et al. [68], who illuminated that tilapia reared under BFT conditions showed upregulated pro-inflammatory cytokines like (TNF-α and IL-1β). Menaga et al. [68] stated that the upregulation of immune and pro-inflammatory related genes modulates the immune response of fish reared in BFT conditions. Some studies have shown that the boost of IL-1β and TNF-α is closely related to the secondary metabolism of microorganisms and the BFT contains many microorganisms [69]. Stressful conditions (e.g., crowding stress and malnutrition) induces negative impacts leading to oxidative stress. It begins with the overproduction of free radicals that damage the immune cells’ DNA and weakens its functions [70]. The present study showed that the antioxidative related indices (SOD, GR, and CAT) were concurrent with the immune-related variables (IL-8, CC-chemokine, and C-X-C chemokine), and both showed enhanced responses in the groups that received glycerol or molasses as carbon sources either under LSD or HSD.

## 5. Conclusions

In conclusion, our study showed that BFT has a highly positive effect on the health condition and immune response of Nile tilapia. Only slight differences were observed amongst the different organic carbon sources treatments. Overall, this study has reported that immune response gene expression is better in low stocking density than high stocking density and is improved by carbon addition. More specifically, based on the overall performances of tilapia reared under low or high stocking density, using molasses is recommended as a carbon source to promote the performances and health status of Nile tilapia cultured in a biofloc system.

## Figures and Tables

**Table 1:** Sequences of primers used in real time PCR.

Genes	Primer Sequence (5′–3′)	Annealing Temp. (°C)	Amplicon (Size pb)	Acc. Number	Reference
IL-8	F: GCACTGCCGCTGCATTAAG	60 °C	128 or 85	DQ061114.1	[34]
	R: GCAGTGGGAGTTGGGAAGAA				
CC-chemokine	F: ACAGAGCCGATCTTGGGTTACTTG	60 °C	228	FF279635.1	[35]
	R: TGAAGGAGAGGCGGTGGATGTTAT				
CXC-chemokine	F: CTATCCATGGAGCCTCAGGT	60 °C	146	XM_00345220	[36]
	R: CTTCTTGAGCGTGGCAATAA				
TLR-7	F: TCAGCAGGGTGAGAGCATAC	63 °C	143	XM_00547 7981.1	[37]
	R: ACATATCCCAGCCGTAGAGG				
β-actin	F: CGAAAGCATTTGCCAAGAAT	60 °C	136	XM_003455949.2	[38]
	R: GGCATCGTTTATGGTCGG				

**Table 2:** Hematological parameters of Nile tilapia juveniles grown at different densities in a conventional intensive culture system and outdoor biofloc technology system (mean ± SD).

Stocking Density	Carbon Source	RBCs (×10^6^/μL)	Hemoglobin (mg/dL)	PCV (%)	WBCs (μL^−1^)	Monocyte (%)	Lympho-Cyte (%)	Eosinophil (%)
Low (140 fish/m^3^)	Control	2.74 ± 0.38 c	8.76 ± 1.78 c	27.8 ± 2.68 c	126,520 ± 18,122.14 a	24.00 ± 7.0 b	5.0 ± 2.24 b	1.6 ± 1.67
	Glycerol	4.19 ± 0.32 a	12.04 ± 0.81 a	46.4 ± 6.80 a	51,200 ± 15,546.70 e	33.4 ± 3.65 a	2.2 ± 0.84 bc	1.0 + 0.71
	Molasses	4.41 ± 0.41 a	12.00 ± 0.79 a	43.4 ± 4.04 ab	78,200 ± 9038.81 cd	34.4 ± 4.98 a	3.0 ± 1.41 bc	0.4 ± 0.55
	Starch	4.03 ± 0.37 a	12.10 ± 0.8 a	40.0 ± 4.95 b	96,840 ± 15,891.13 bc	34.0 ± 3.81 a	2.0 ± 1.22 c	1.0 ± 0.71
High (280 fish/m^3^)	Control	2.50 ± 0.32 c	8.78 ± 0.95 c	25.8 ± 3.63 c	107,200 ± 21,545.30 ab	22.8 ± 1.92 b	9.4 ± 4.39 a	1.0 ± 0.71
	Glycerol	3.42 ± 0.21 b	10.42 ± 0.56 b	41.6 ± 2.79 ab	74,200 ± 13,007.69 cde	35.4 ± 4.04 a	2.8 ± 1.10 bc	1.4 ± 0.89
	Molasses	4.46 ± 0.32 a	12.66 ± 0.8 a	43.8 ± 4.21 b	67,580 ± 4934.78 e	35.2 ± 3.11 a	2.6 ± 0.89 bc	1.2 ± 0.84
	Starch	3.53 ± 0.37 b	11.02 ± 0.56 b	38.8 ± 2.39 ab	95,000 ± 33,600.60 bc	33.2 ± 6.57 a	3.0 ± 1.41 bc	0.6 ± 0.55

Different letters within the same column are significantly different at *p* < 0.05.

**Table 3:** Serum biochemical parameters of Nile tilapia juveniles grown at different densities in a conventional intensive culture system and outdoor biofloc technology system (mean ± SD).

Stocking Density	Carbon Source	Protein (g/dL)	Albumin (g/dL)	Globulin (g/dL)	A/G Ratio	Glucose (mg/dL)	Cortisol (ng/dL)
Low (140 fish/m^3^)	Control	4.16 ± 0.47 cd	1.37 ± 0.30 bc	2.79 ± 0.30 cd	0.50 ± 0.10	144.4 ± 19.68 a	41.00 ± 5.57 a
	Glycerol	4.94 ± 0.59 b	1.41 ± 0.10 bc	3.53 ± 0.58 ab	0.41 ± 0.07	75.0 ± 17.32 cd	6.00 ± 0.85 e
	Molasses	4.97 ± 0.27 b	1.39 ± 0.03 bc	3.59 + 0.26 ab	0.39 ± 0.03	73.6 ± 7.13 cd	12.04 ± 2.74 de
	Starch	4.87 ± 0.47 bc	1.56 ± 0.15 ab	3.31 ± 0.42 abcd	0.478 ± 0.07	94.6 ± 14.31 bc	27.16 ± 3.82 c
High (280 fish/m^3^)	Control	3.82 ± 0.74 d	1.19 ± 0.30 c	2.63 ± 0.78 d	0.48 ± 0.18	139.8 ± 13.97 a	37.6 ± 10.53 a
	Glycerol	4.36 ± 0.32 bcd	1.38 ± 0.14 bc	3.12 ± 0.26 bcd	0.4 ± 0.05	67.20 ± 8.20 d	8.36 ± 4.58 e
	Molasses	4.91 ± 0.81 bc	1.47 ± 0.07 bc	3.45 ± 0.77 abc	0.44 ± 0.07	78.0 ± 10.68 bcd	17.52 ± 2.05 d
	Starch	5.73 ± 0.42 a	1.79 ± 0.28 a	3.94 ± 0.25 a	0.45 ± 0.07	97.8 ± 27.46 b	32.40 ± 6.39 bc

Different letters within the same column are significantly different at *p* < 0.05.

**Table 4:** Antioxidant levels of *Nile tilapia* juveniles grown at different densities in a conventional intensive culture system and outdoor biofloc technology system (mean ± SD).

Stocking Density	Carbon Source	Superoxide Dismutase (U/L)	Nitric Oxide (U/L)	Glutathione Reductase (U/L)	Catalase (U/L)
Low (140 fish/m^3^)	Control	33.8 ± 7.26 cd	23.52 ± 6.36 a	27.2 ± 2.77 cd	27.6 ± 3.44 de
	Glycerol	62.4 ± 9.79 a	7.72 ± 1.29 d	43.4 ± 7.40 a	44.6 ± 4.28 a
	Molasses	34.8 ± 7.79 cd	15.24 ± 2.31 bc	31.4 ± 6.80 bcd	31.4 ± 2.41 cde
	Starch	31.6 ± 5.77 cd	17.12 ± 4.16 b	33.2 ± 4.66 bc	33.4 ± 3.91 bcd
High (280 fish/m^3^)	Control	26.0 ± 3.39 d	24.40 ± 7.84 a	24.2 ± 6.61 d	19.4 ± 5.22 f
	Glycerol	48.4 ± 10.11 b	10.28 ± 1.64 cd	36.2 ± 5.40 b	38.2 ± 3.56 b
	Molasses	39.8 ± 3.70 bc	16.06 ± 2.58 b	34.8 ± 4.44 bc	35.2 ± 4.02 bc
	Starch	33.6 ± 5.13 cd	17.52 ± 2.23 b	31.0 ± 3.81 bcd	26.6 ± 7.20 e

Different letters within the same column are significantly different at *p* < 0.05.

## Data Availability

Not applicable.
